# ATTITUDES TOWARDS AGING PERCEPTIONS OF AGING AND HEALTH OF OLDER ADULT SPOUSES

**DOI:** 10.1093/geroni/igz038.1700

**Published:** 2019-11-08

**Authors:** Jennifer L Smith, Joseph G Bihary, Dugan O’Connor, Ajla Basic, Cate O'Brien

**Affiliations:** Mather LifeWays Institute on Aging, Evanston, Illinois, United States

## Abstract

Evidence suggests that negative perceptions of aging are associated with worse health outcomes. Research also shows that marital partners can mutually influence each other’s health; however, limited research has explored perceptions of aging within couples. The purpose of the current study was to examine the association between self and partner perceptions of aging and health among married older adult couples. Both members of 452 couples (average age: wives = 80.6 years, husbands = 82.7 years) completed a survey that measured perceptions of aging and health (i.e., self-reported health, healthy diet, physical functioning, stress, and physical activity). Data were analyzed using actor-partner interdependence models, and analyses were conducted separately for positive and negative perceptions of aging, controlling for self and partner’s age, depression, and chronic health conditions as well as gender and income. Consistent with previous research, analyses revealed that one’s own positive perceptions of aging were related to better health across all measures, and the opposite pattern was found for negative perceptions of aging. An examination of partner effects revealed that people reported better physical functioning and greater physical activity when their spouses had more positive perceptions of aging. Similarly, husbands reported less stress when wives had more positive perceptions of aging, but there was not a partner effect for wives. In contrast, people reported lower subjective health and less healthy diets when their spouses had more negative perceptions of aging. These findings suggest that health-promotion efforts should consider partners’ perceptions of aging as a potential resource or risk factor.

